# NADPH oxidase promotes Parkinsonian phenotypes by impairing autophagic flux in an mTORC1-independent fashion in a cellular model of Parkinson’s disease

**DOI:** 10.1038/srep22866

**Published:** 2016-03-10

**Authors:** Rituraj Pal, Lakshya Bajaj, Jaiprakash Sharma, Michela Palmieri, Alberto Di Ronza, Parisa Lotfi, Arindam Chaudhury, Joel Neilson, Marco Sardiello, George G. Rodney

**Affiliations:** 1 Department of Molecular Physiology and Biophysics, Baylor College of Medicine, Houston, United States; 2 Department of Molecular and Human Genetics, Baylor College of Medicine, Houston, United States

## Abstract

Oxidative stress and aberrant accumulation of misfolded proteins in the cytosol are key pathological features associated with Parkinson’s disease (PD). NADPH oxidase (Nox2) is upregulated in the pathogenesis of PD; however, the underlying mechanism(s) of Nox2-mediated oxidative stress in PD pathogenesis are still unknown. Using a rotenone-inducible cellular model of PD, we observed that a short exposure to rotenone (0.5 μM) resulted in impaired autophagic flux through activation of a Nox2 dependent Src/PI3K/Akt axis, with a consequent disruption of a Beclin1-VPS34 interaction that was independent of mTORC1 activity. Sustained exposure to rotenone at a higher dose (10 μM) decreased mTORC1 activity; however, autophagic flux was still impaired due to dysregulation of lysosomal activity with subsequent induction of the apoptotic machinery. Cumulatively, our results highlight a complex pathogenic mechanism for PD where short- and long-term oxidative stress alters different signaling pathways, ultimately resulting in anomalous autophagic activity and disease phenotype. Inhibition of Nox2-dependent oxidative stress attenuated the impaired autophagy and cell death, highlighting the importance and therapeutic potential of these pathways for treating patients with PD.

Reactive oxygen species (ROS) play pivotal roles in regulating signaling molecules, but when in excess they induce oxidative stress; which has been implicated as a key pathological factor in “sporadic” forms of Parkinson’s disease (PD) and other neurodegenerative diseases. Environmental toxins, such as rotenone, have been well-established as causal agents of sporadic form of PD due to its ability to generate reactive oxygen and nitrogen species (ROS/RNS)^1^. Previous work has shown that oxidative stress impairs autophagic flux and decreases lysosomal biogenesis and function in animal models of PD^2^,^3^. Up-regulation of NADPH oxidase (Nox2), a major superoxide-producing enzyme complex^4^, has recently been associated with PD pathogenesis in human patients and animal models^5^. Shacka and colleagues suggest that rotenone-induced oxidative stress impairs autophagic flux and promotes accumulation of protein aggregates in an *in vitro* model of PD^6^. We and others have recently demonstrated the ability of rotenone to induce oxidative stress via activation of the Nox2 complex^7^,^8^,^9^. We have also shown that enhanced Nox2-dependent ROS production drives impaired autophagic flux and lysosomal dysfunction by activation of Src/PI3K/mTORC1 pathway in a mouse model of muscle degeneration^10^. The classical paradigm of autophagy in mammalian cells involves ULK1, a key pro-autophagy adapter kinase essential to the nucleation of the autophagophore membrane. Activation of the serine/threonine kinase mTORC1 inhibits autophagy through phosphorylation of ULK1 at S757, thus subsequent inhibition of ULK1 activity^11^. The energy sensitive AMP activated protein kinase (AMPK) promotes autophagy, in an mTORC1-independent manner, by directly activating ULK1 through phosphorylation of ULK1 at S317^12^. Therefore, we investigated whether rotenone impairs autophagic flux and lysosomal activity through a Nox2/Src/PI3K/mTORC1/ULK1- dependent pathway or via AMPK/ULK1 pathway, leading to accumulation of protein aggregates in PD pathogenesis.

Using the human neuroblastoma SHSY-5Y cell line we investigated the molecular mechanisms by which rotenone-dependent activation of Nox2 leads to alterations in the homeostasis of autophagy. We found that rotenone (0.5 μM) for a short period of time, characterized as “mild activation”, increased Nox2-ROS generation and impaired autophagic flux in an mTORC1/AMPK-independent manner by inducing Src/PI3K/Akt-mediated phosphorylation of Beclin1 at S295 and concomitant disruption of Beclin1-VPS34 complex. We have also shown that exposure to a higher concentration of rotenone (10 μM) for longer duration, characterized as “excessive activation”, induced further increase in ROS generation, resulting in a decrease in lysosomal acidification, lysosomal activity and autophagic flux. Notably, inhibition of Nox2-dependent oxidative stress in either case significantly rescued homeostasis of autophagic flux. Taken together, our findings unravel a novel mechanism by which Nox2-dependent oxidative stress leads to the pathogenesis of PD.

## Results

### Mild activation of Nox2 increases ROS generation, impairs autophagic flux and induces protein accumulation

**Figure 1:**
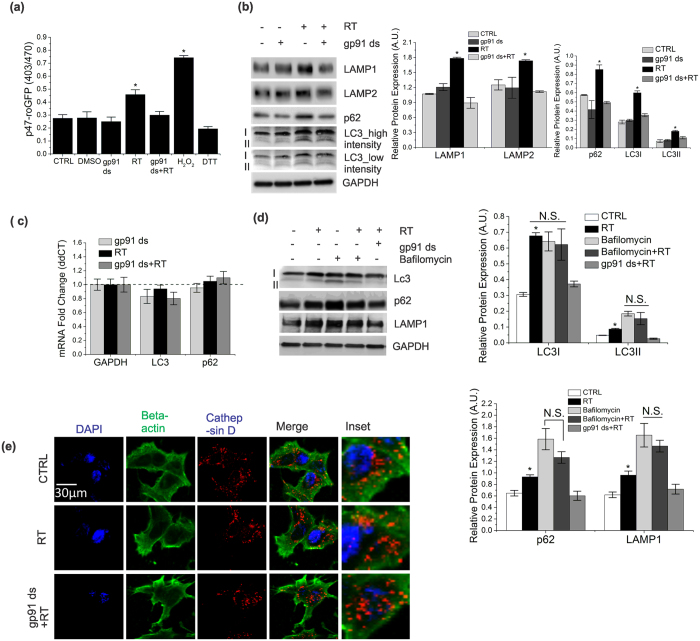
Mild activation of Nox2 increases ROS generation impairs autophagic flux and induces protein accumulation. (**a**) SHSY-5Y cells were transfected with p47-roGFP and incubated 24 h, followed by treatment with 0.5 μM rotenone or DMSO (0.1%) for 6 h. Some cells were preincubated with 5 μM gp91 ds for 1 h followed by 6 hours of rotenone treatment. As control, some cells were treated with 5 μM gp91 ds for 7 h. ROS generation was measured by monitoring p47-roGFP fluorescence intensity. Hydrogen peroxide (H_2_O_2,_ 200 μM for 15 min) and Dithiothreitol (DTT, 10 mM for 15 min) were used as controls to obtain maximum (oxidation) and minimum (reduction) levels. (**b**) Lysates from the cells treated as in (**a**) were analyzed by immunoblotting with antibodies as indicated. (**c**) mRNA levels of LC3 and p62 were measured by qRT-PCR analysis. Results are normalized to GAPDH. Dotted line indicates results from DMSO (vehicle, 0.1%) treatment. (**d**) To assess autophagic flux, cells were treated with gp91 ds and rotenone in a similar fashion to (**a**). Bafilomycin A (160 nM) was added to the cells 4 h prior to lysis. LAMP1, LC3 and p62 levels were analyzed by immunoblotting. (**e**) Cells were treated as in (**a**) and were labeled with endogenous beta-actin (green) and cathepsin D (red) for immunofluorescence. Representative cells are shown. The punctate structures indicate lysosomal localization of cathepsin D. Nucleus was labeled with DAPI (blue). GAPDH was detected as a loading control for all immunoblots. All bar diagrams indicate quantitative analysis of at least 3 biological replicates. Results are represented as means of SE (SEM). *p < 0.05 versus all groups, unless otherwise indicated. N.S. indicates non-significant.

To investigate the early impact of Nox2-dependent oxidative stress on autophagy in a model of PD, we treated SHSY-5Y cells^1^ with 0.5 μM of rotenone for 6 h. ROS generation was measured using a Nox2-specific redox sensor (p47-roGFP)^4^ and a general ROS sensor (DCF-DA). Rotenone-treated cells showed ~2 fold increase in ROS generation compared to untreated cells, which was suppressed by the Nox2-specific inhibitor, gp91 ds (Fig. 1a & supplementary Figure S1a). Immunoblot analysis showed a robust increase in steady state expression of LC3 (LC3I and LC3II), LAMP1&2, and p62 upon rotenone treatment compared to untreated cells, which were attenuated by pre-incubation with gp91 ds (Fig. 1b), indicating that the effect on autophagy was being mediated by Nox-2 dependent ROS. Rotenone treatment did not significantly alter the mRNA levels of *LC3* or *p62* (Fig. 1c), indicating that the Nox2-dependent increase in the levels of LC3 and p62 proteins is not due to an increase in their transcription. To examine whether the increased protein level is a result from impairment in autophagic flux, we inhibited LC3-II/autophagosome degradation with bafilomycin A_1_ (hereafter referred to as “bafilomycin”), a well-established method for monitoring autophagic flux^13^,^14^. We found that rotenone increased LC3, LAMP1 and p62 protein levels to the same degree as bafilomycin, with no synergistic effect when added in combination (Fig. 1d). These data suggested that LC3, p62 and LAMP accumulate in the cytosol as a result of impaired autophagic flux. To determine whether the impairment of autophagic flux is associated with lysosomal membrane permeabilization (LMP), and subsequent reduction in lysosomal function, we assessed cathepsin D localization. During LMP, cathepsin D translocates from the lysosomal lumen (punctate structure) to the cytosol (diffuse pattern)^3^. We did not detect any changes in punctate structure of cathepsin D upon rotenone treatment (Fig. 1e). Furthermore, we found no significant change in cell survivability (Supplementary Figure S1b) in response to 0.5 μM rotenone for 6 h. These data imply that impairment of autophagic flux by mild oxidative stress is independent of lysosomal activity without leading to cell death.

### Mild activation of Nox2 impairs autophagic flux in mTORC1/AMPK-independent manner

**Figure 2:**
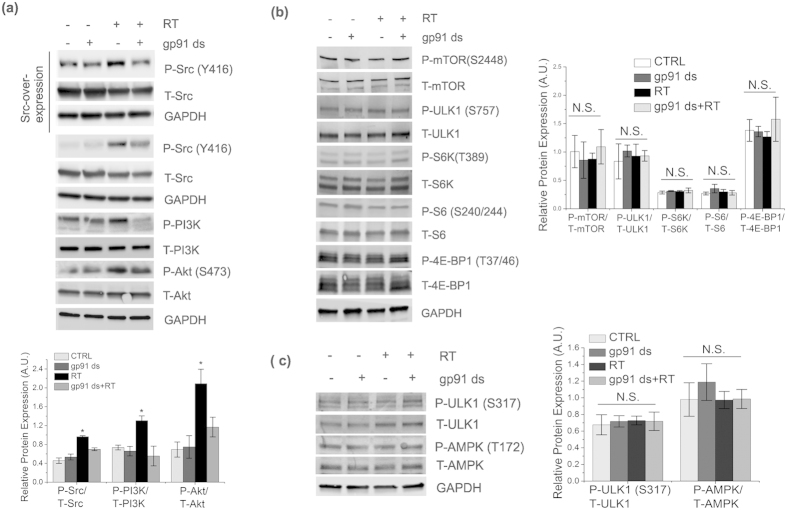
Mild activation of Nox2 impairs autophagic flux in mTORC1/AMPK-independent manner. (**a–c**) SHSY-5Y cells were treated or untreated with 0.5 μM rotenone for 6 h. Some cells were preincubated with 5 μM gp91 ds for 1 h prior to the 6 h of rotenone treatment. As controls, some cells were treated with 5 μm gp91 ds for 7 h. Then, cells were lysed and analyzed by immunoblotting with indicated antibodies. Wild-type (WT-Src) c-Src construct (Addgene plasmid # 17672) was used for over expression of Src kinase in (**a**). GAPDH was detected as a loading control for all immunoblots. Bar diagram indicates quantitative analysis of at least 3 biological replicates. Results are represented as means of SE (SEM). *p < 0.05 versus all groups, unless otherwise indicated. N.S. indicates non-significant.

ROS generation has been shown to activate the Src/PI3K/Akt pathway^15^,^16^, a major regulatory pathway for mTORC1 activation followed by inactivation of ULK1 and suppression of autophagy^10^,^17^,^18^. Therefore, we tested whether a Src/PI3K/Akt/mTORC1/ULK1 pathway is mediating impaired autophagic flux. Rotenone-treated cells showed a significant increase in phosphorylation of Src, PI3K, and Akt compared to untreated cells, which was suppressed upon inhibition of Nox2 (Fig. 2a). We did not observe concurrent activation beyond basal levels of mTORC1 or AMPK (Fig. 2b,c). Rotenone-induced activation of Src was significantly rescued by preincubation with either gp91 ds or by the general ROS scavenger, NAC (Supplementary Figure S1c), suggesting that Nox2-mediated ROS generation induces activation of Src kinase. Together, our results indicate that mild activation of Nox2 for a short period of time dysregulates autophagic machinery via an mTORC1-independent pathway.

### Mild activation of Nox2 impairs autophagic flux by disrupting Beclin1-VPS34 autophagy initiation complex

**Figure 3:**
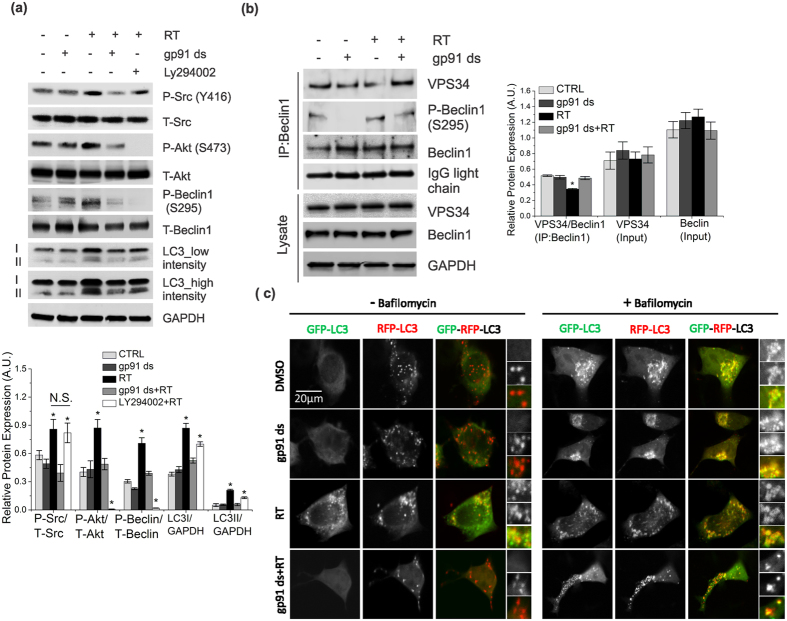
Mild activation of Nox2 impairs autophagic flux by disrupting Beclin1-VPS34 autophagy initiation complex. (**a**) SHSY-5Y cells were treated or untreated with 0.5 μM rotenone for 6 h. Some rotenone-treated cells were treated with 50 μM LY294002 prior (2 h) to lysing of the cells. Lysates were analyzed by immunoblotting with antibodies as indicated. (**b**) Cells were treated or untreated with 0.5 μM rotenone for 6 h. Some cells were preincubated with 5 μM gp91 ds for 1 h prior to the 6 h of rotenone treatment. As controls, some cells were treated with 5 μm gp91 ds for 7 h. Cells were then lysed and immunoprecipitated with a Beclin1 antibody. Immunoblots of IPs or cell lysates were probed with the indicated antibodies. (**c**) Cells were transfected with GFP-RFP-LC3 construct and incubated for 24 h, followed by treatment as indicated in (1a). Bafilomycin was used as a positive control of autophagic flux blocker. Representative cells are shown where yellow pixels indicate colocalization of GFP and RFP in the merged images. Nucleus is indicated with DAPI (blue) staining. GAPDH was detected as a loading control for all immunoblots. Bar diagram indicates quantitative analysis of at least 3 biological replicates. Results are represented as means of SE (SEM). *p < 0.05 versus all groups, unless otherwise indicated. N.S. indicates non-significant.

Beclin1 regulates the initiation of autophagosome formation as a part of the hVps34/PI3K complex^19^. Rotenone treated cells showed a significant increase in phosphorylation of Beclin1 (S295), which was decreased upon inhibition of either Nox2 or PI3K by gp91 ds and LY294002, respectively (Fig. 3a). Next, we tested whether Nox2-dependent phosphorylation of Beclin1 disrupts Beclin1-VPS34 interaction. Immunoprecipitation analysis showed that rotenone decreased the interaction between Beclin1 and VPS34, which was significantly rescued upon inhibition of Nox2 (Fig. 3b). We then tested whether Nox2-dependent disruption of Belcin1 interaction with VPS34 led to a decrease in autophagolysosome formation by using tandem GFP-RFP-LC3 construct (Fig. 3c). We found a significant decrease in red LC3-puncta in rotenone treated cells compared to control cells, with no change in lysosomal activity (Fig. 1e), suggesting an impairment in autophagolysosome formation by rotenone-induced activation of Nox2. Moreover, inhibition of Nox2-ROS by gp91 ds significantly rescued autophagolysosome formation (Fig. 3c), as evidenced by increased red LC3 puncta. Post-treatment with bafilomycin blocked autophagolysosome formation, resulting in yellow puncta of GFP-RFP-LC3 (Fig. 3c).

### Activation of Src kinase dysregulates autophagolysosome formation by disrupting Beclin1-VPS34 complex

**Figure 4:**
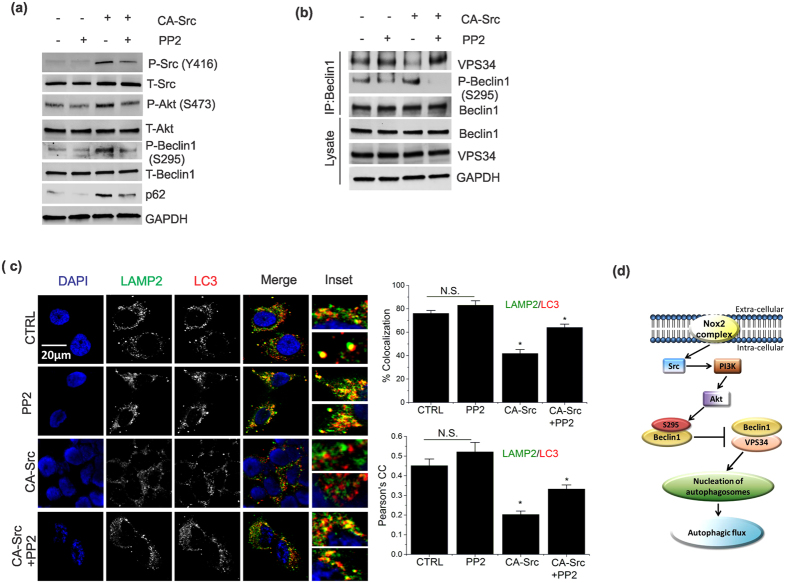
Activation of Src kinase dysregulates autophagolysosome formation by disrupting Beclin1-VPS34 complex. (**a**) To determine the effect of activation of Src on autophagic flux, cells were transfected with constitutively active Src kinase (CA-Src, addgene plasmid # 13660) or left untransfected for 24 h. Cells treated or untreated with 10 μM PP2 prior (2 h) to lysis. Cell lysates were analyzed by immunoblotting with the antibodies as indicated. (**b**) Cells treated as in (**a**) were lysed and immunoprecipitated with Beclin1 antibody. Immunoblots of IPs or cell lysates were probed with the indicated antibodies. (**c**) Cells were treated as in (**a**) prior to immunofluorescent labeling of endogenous LAMP2 (green) and LC3B (red). Representative cells are shown where yellow pixels indicate colocalization in the merged images. Nucleus is indicated with DAPI (blue) staining. Percent colocalization and Pearson’s correlation coefficient (PCC) are graphed as a mean ± SEM. GAPDH was detected as a loading control for immunoblots. Bar diagram indicates quantitative analysis of at least 3 biological replicates. Results are represented as means of SE (SEM). *p < 0.05 versus all groups, unless otherwise indicated. (**d**) Model shows activated Nox2 complex impairs autophagic flux by disrupting Beclin1-VPS34 interaction *via* activation of Akt.

To assess the role of Src kinase in Akt/Beclin1-dependent impairment of autophagic flux, cells were transfected with a constitutively active-Src (CA-Src). CA-Src transfection resulted in a robust increase in phosphorylation of Src, Akt and Beclin1, which was significantly blocked by the inhibition of Src kinase with Src inhibitor, PP2 (Fig. 4a). Concomitant with increased phosphorylation of Beclin1 at S295, autophagic flux was decreased as evidenced by increased steady state expression of p62 (Fig. 4a). CA-Src attenuated initiation of autophagy by disrupting Beclin1-VPS34 interaction (Fig. 4b), resulting in decreased autophagolysosome formation (Fig. 4c). Together, these results indicate that rotenone (0.5 μM) results in a mild activation of Nox2, which leads to impairment of autophagic flux by inhibiting the maturation process of autophagosomes (see model in Fig. 4d).

**Figure 5:**
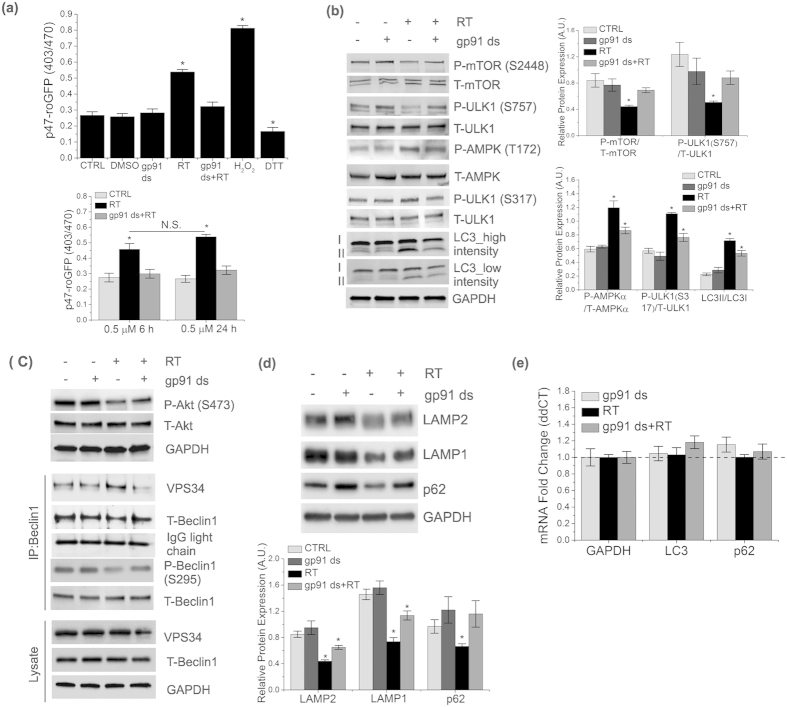
Prolonged rotenone exposure triggers autophagy signaling pathways in SHSY-5Y cells. (**a**) SHSY-5Y cells were transfected with p47-roGFP and incubated for 24 h, followed by treatment with 0.5 μM rotenone or DMSO (0.1%) for 24 h. Some cells were preincubated with 5 μM gp91 ds for 1 h followed by 6 h of rotenone treatment. As control, some cells were treated with 5 μM gp91 ds for 25 h. All other treatments and measurements are similar to (1a). (**b–d**) Lysates from cells treated as in (**a**) were analyzed by immunoblot analysis using the antibodies as indicated. For immunoprecipitation (IP), cells were treated as indicated in (**a**) prior to lysis and immunoprecipitation with a Beclin1 antibody. Immunoblots of IPs or cell lysates were probed with the indicated antibodies. (**e**) mRNA levels of LC3 and p62 were measured by qRT-PCR analysis. Results are normalized to GAPDH, which was used as a control. Dotted line indicates results from DMSO (vehicle, 0.1%) treatment. GAPDH was detected as a loading control for all immunoblots. Bar diagram indicates quantitative analysis of at least 3 biological replicates. Results are represented as means of SE (SEM) *p < 0.05 versus all groups, unless otherwise indicated.

We then investigated whether 0.5 μM rotenone treatment for a longer duration (for 24 h) could further increase Nox2-dependent oxidative stress and worsen the autophagic machinery. Interestingly, we found no further increase in Nox2-specific ROS generation, as there is no significant change in p47-roGFP fluorescence intensity between 6 h and 24 h of rotenone treatment (Fig. 5a). Unexpectedly, we found that rotenone treated cells showed a significant decrease in mTORC1 activity, an increase in the phosphorylation of AMPK and ULK1 (S275 and S317) and an increase in the LC3II/LC3I ratio, all of which were significantly rescued upon inhibition of Nox2 (Fig. 5b), suggesting an increase in autophagy signaling. We have also found a significant decrease in phosphorylation of Akt and beclin1 (Fig. 5c), and concomitant increase in the interaction of beclin1 with VPS34 in response to rotenone (Fig. 5c), which were reversed upon inhibition of Nox2. Furthermore, we found a dramatic increase in autophagic flux as evidenced by decrease in the levels of LAMPs and p62, which were significantly rescued upon inhibition of Nox2 (Fig. 5d). There was no significant effect of rotenone on mRNA levels of LC3 or p62, indicating that Nox2-dependent alterations in protein levels of LC3 and p62 are not due to a decrease at the transcriptional level (Fig. 5e). We also observed no changes in lysosomal activity as evidenced by an unaltered punctate structure of the lysosomal hydrolase, cathepsin D (Supplementary Figure S2a). Our results indicate a mild increase in delivery or fusion of autophagosomes to lysosome, as evidenced by a mild increase in red puncta of GFP-RFP-LC3 (Supplementary Figure S2b). We did not observe a significant alteration in cell survivability (Supplementary Figure S2c). Collectively, these results demonstrate that 0.5 μM rotenone for longer exposure triggers the activation of autophagy signaling pathways.

### Excessive activation of Nox2 further increases ROS production and protein accumulation

**Figure 6:**
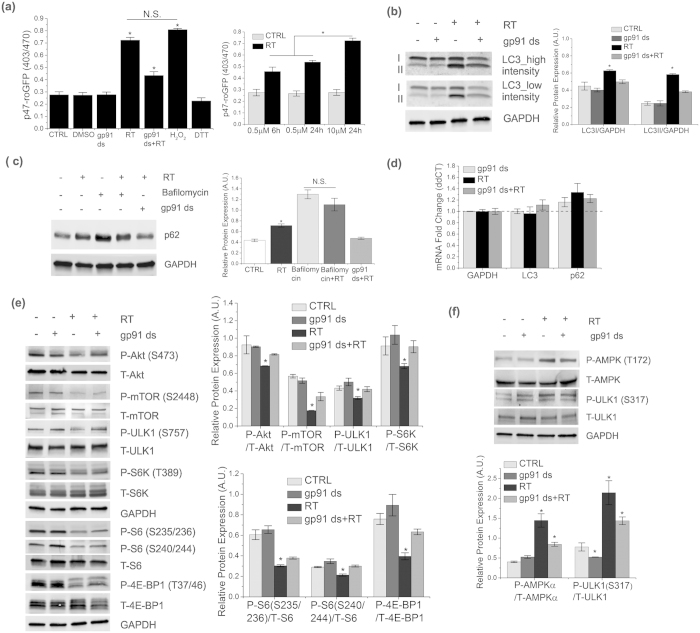
Excessive activation of Nox2 further increases ROS production and protein accumulation. (**a**) SHSY-5Y cells were transfected with p47-roGFP and incubated for 24 h, followed by treatment with 10 μM rotenone or DMSO (0.1%)for 24 h. Some cells were preincubated with 5 μM gp91 ds for 1 h followed by 6 h of rotenone treatment. As control, some cells were treated with 5 μM gp91 ds for 25 h. All other treatments and measurements are similar to (1a). (**b**) Cells were treated or untreated with 10 μM rotenone for 24 h. Some cells were preincubated with 5 μM gp91 ds for 1 h prior to the 24 h of rotenone treatment. As controls, some cells were treated with 5 μm gp91 ds for 25 h. Proteins were extracted and lysates were analyzed by immunoblotting with the indicated antibodies. (**c**) Autophagic flux was detected in the cells treated with gp91 ds and rotenone in a similar fashion to (**c**). Bafilomycin A was added to the cells 4 h prior to lysis. p62 protein levels were analyzed by immunoblotting. (**d**) mRNA levels of LC3 and p62 were measured by qRT-PCR analysis. Results are normalized to GAPDH, which was used as a control. Dotted line indicates results from DMSO (vehicle, 0.1%) treatment. (**e,f**) Lysates from cells treated as in (**c**) were analyzed by using immunoblot assay with the antibodies as indicated. GAPDH was detected as a loading control for immunoblots. Bar diagram indicates quantitative analysis of at least 3 biological replicates Results are represented as means of SE (SEM). *p < 0.05 versus all groups, unless otherwise indicated. N.S. indicates non-significant.

Previous studies have identified accumulation of autophagic vacuoles and neuronal cell death in response to high doses of rotenone^6^,^20^. To determine whether these abnormalities are Nox2 dependent, we treated the cells with 10 μM rotenone for 24 h. We found a significant increase in ROS generation compared to untreated cells (~3.5 fold, Fig. 6a & Supplementary Figure S3a) and 0.5 μM rotenone exposure (Fig. 5a). We found that gp91 ds abolished rotenone-induced increased fluorescence of p47-roGFP, while DCF fluorescence was partially, but not completely, inhibited by gp91 ds (Supplementary Figure S3a). These results suggest that 10 μM rotenone for 24 h stimulates ROS generation not only through Nox2, but possibly from mitochondria. Immunoblot analysis showed a robust increase in LC3 (LC3I&II) levels in rotenone treated cells (Fig. 6b). We then tested the homeostasis of autophagic flux upon rotenone-mediated excessive activation of Nox2. Consistent with previous studies^6^,^20^, we also observed an increase in p62 levels in response to rotenone treatment, which was partially attenuated by pre-incubation with gp91 ds (Fig. 6c), suggesting a Nox2-dependent blockade of autophagic flux. Post-treatment with bafilomycin failed to further increase the level of p62 in rotenone treated cells compared to the cells treated with bafilomycin alone, indicating that the increase in autophagic vacuoles results from the lack of their degradation from the cytosol (Fig. 6c). There was no significant effect of rotenone on mRNA levels of LC3 or p62, indicating that Nox2-dependent alterations in protein levels of LC3 and p62 are not due to a decrease in transcription (Fig. 6d). Next, we tested whether impairment of autophagy is a result from inhibition of ULK1-activity through either an mTORC1 or AMPK pathway.We found an increase in ULK1 activity (decreased P-S757) due to a significant decrease in Akt/mTORC1 pathway (Fig. 6e). Interestingly, we also found an AMPK-dependent activation of ULK1 (increased P-S317) in response to rotenone treatment (Fig. 6f). Activation of ULK1 by both pathways was prevented upon inhibition of Nox2 (Fig. 6e,f). Consistent with the effect of gp91 ds on ROS generation, we found that gp91 ds significantly, but not completely, rescued the downstream effect of Nox2 activation. These results suggest that excessive Nox2 activation impairs autophagic flux in an mTORC1-independent fashion.

### Excessive activation of Nox2 impairs autophagic flux by decreasing lysosomal function, and induces cell death

**Figure 7:**
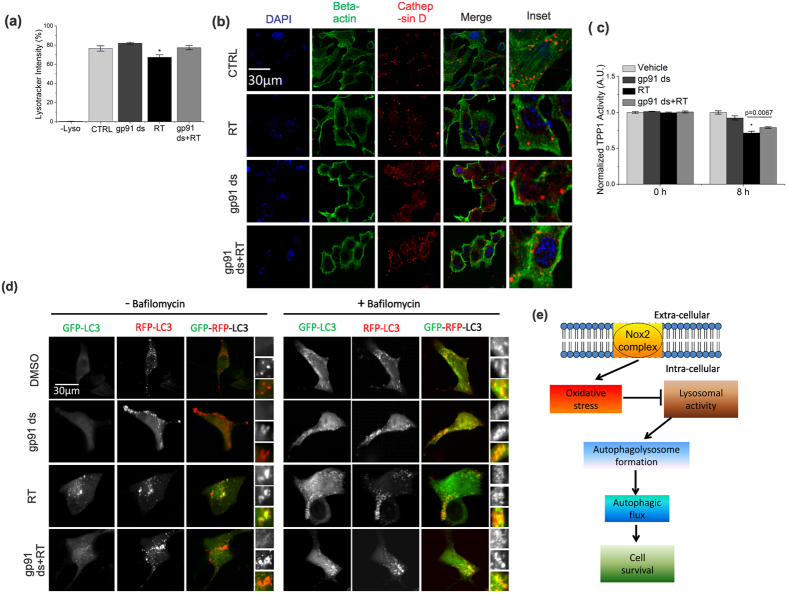
Excessive activation of Nox2 impairs autophagic flux by decreasing lysosomal function, and induces cell death. (**a**) Acidic lysosomal vesicles were measured using lysotracker. SHSY-5Y cells were treated or untreated with 10 μM rotenone for 24 h. Some cells were preincubated with 5 μM gp91 ds for 1 h prior to the 24 h of rotenone treatment. As controls, some cells were treated with 5 μm gp91 ds for 25 h. Cells were stained with lysotracker red DND-99 (200 nM, 30 min) and intensity was measured by flow cytometry. See “materials and methods” for details. (**b**) Cells were treated as in (**a**) prior to immunofluorescent labeling of endogenous beta-actin (green) and cathepsin D (red). Representative cells are shown. The punctate structures indicate lysosomal localization of cathepsin D. Nucleus was labeled with DAPI (blue). (**c**) Lysosomal hydrolase, TPP1, enzyme activity was assessed in cells treated as in (**a**). Results are graphed as a mean ± SEM. *p < 0.05 versus all groups at 8 h. (**d**) Cells were transfected with GFP-RFP-LC3 construct and incubated for 24 h, followed by treatment as indicated in (**a**). Live cell imaging was performed using confocal microscope. Bafilomycin was used as a positive control of autophagic flux blocker. Representative cells are shown where yellow pixels indicate colocalization of GFP and RFP in the merged images. Nucleus is indicated with DAPI (blue) staining. (**e**) Model of the Nox2-dependent impairment of lysosomal activity and autophagic flux. Bar diagram indicates results as mean ± SEM. *p < 0.05 versus all groups.

To explore the molecular mechanisms and pathogenic significance of Nox2-dependent impairment of autophagic flux, we investigated whether activation of Nox2 inhibits the degradation of autophagolysosomes by disrupting lysosomal function, and subsequent impairment in autophagolysosome formation. To determine the effect of Nox2-dependent oxidative stress on lysosomal activity, we analyzed the presence of the acidic vesicles utilizing flow cytometry and the acidotropic dye, lysotracker red (LTR) (Fig. 7a). Similar to previous findings^6^, we found that cells treated with rotenone showed a significant decrease in LTR mean fluorescence intensity compared to untreated cells. We now provide mechanistic details on this rotenone induced alteration of lysosomal function, as cells pre-incubated with gp91 ds significantly rescued the rotenone-induced decrease in LTR fluorescence (Fig. 7a). These results suggest that Nox2-dependent oxidative stress possibly deregulates lysosomal pH. In addition, we found that cells treated with rotenone showed a mild decrease in punctate form and concomitant increase in diffused form of cathepsin D (Fig. 7b). The enzymatic activity of lysosomal hydrolase, TPP1, in response to rotenone treatment was lower compared to untreated cells (Fig. 7c). Rotenone induced alterations in cathepsin D localization and TPP1 activity was significantly rescued by inhibition of Nox2. We then tested whether a decrease in lysosomal activity could lead to the impairment in autophagolysosome formation using tandem GFP-RFP-LC3. We found a significant decrease in red LC3-puncta in rotenone treated cells compared to control cells (Fig. 7d), suggesting impairment in delivery or fusion of autophagosomes to lysosomes. Furthermore, excessive activation of Nox2 triggered an increase in apoptotic signaling, as evidenced by an increase in expression levels of cleaved PARP-1 and cleaved caspase3 (Supplementary Figure 3b). More importantly, cells preincubated with gp91 ds showed a significant protection against rotenone-dependent upregulation in apoptotic signaling (Supplementary Figure 4b). We have also found that rotenone treated cells showed a decrease in cell survivability compared to control cells, which was partially rescued by inhibition of Nox2 (Supplementary Figure 3c). Together, these results demonstrate that rotenone mediated excessive activation of Nox2 dysregulates lysosomal function, thereby impairing autophagic flux and inducing cell death (See model in Fig. 7e).These results highlight the involvement of Nox2-dependent oxidative stress in apoptotic cell death. Future studies are warranted to delineate the association between Nox2-dependent impaired autophagic flux and apoptotic cell death.

## Discussion

Autophagy is a dynamic cellular pathway involved in the degradation of aggregates of misfolded protein and other cellular constituents^21^. Aberrant aggregate formation has been associated with impairment of autophagic flux, leading to neurodegeneration^22^. The classical paradigm of autophagy in mammalian cells involves ULK1, a key pro-autophagy adapter kinase essential to the nucleation of the autophagophore membrane. While activation of ULK1 by either mTORC1 or the energy sensitive AMPK promotes autophagy^11^,^12^, activation of Akt has been shown to impair autophagic flux in an mTORC1-independent fashion^23^. Akt-mediated phosphorylation of Beclin1 at S295 leads to immature autophagosome formation, resulting in an accumulation of ubiquitin-interacting protein p62 (SQSMT1), which is a hallmark of impaired autophagic flux^24^. In this study, we have determined that oxidative stress, caused by rotenone-dependent activation of Nox2-complex, results in neuronal cell death by complex dose and time dependent mechanisms. Rotenone-dependent mild activation of Nox2 increases ROS production, leading to dysregulation of autophagic flux and accumulation of proteins in the cytosol in an mTORC1-independent fashion. Previously, both *in vitro* and *in vivo* studies have shown that deficiency in lysosomal hydrolase, cathepsin D, hinders clearance of alpha-synuclein aggregates, thereby promoting alpha-synuclein toxicity, a hallmark of PD pathogenesis^25^,^26^. Here, we have found that excessive activation of Nox2 by rotenone, with high dose and a longer period of time, increases lysosomal pH; thereby decreasing lysosomal activity, impairing autophagic flux and impairing clearance of protein aggregates. Together, these studies strongly suggest impairment in lysosomal degradation in PD pathogenesis.

Recent studies have also evidenced that accumulation of protein aggregates is a proximal trigger for the upregulation of basal autophagy signaling pathways, thereby attempting to maintain the homeodynamics by the clearance of the accumulated proteins from the cytosol^27^,^28^. Thus, increased ULK1-activity through TORC1 or AMPK in response to prolonged rotenone exposure could be due to a feed-back response to accumulated proteins in the cytosol caused by the early decrease in autophagic flux. We found that inhibition of rotenone-induced early activation of Nox2-ROS significantly prevented the effects of prolonged rotenone exposure (induction of autophagy signaling) . Our findings indicate that prolonged exposure to ROS induces autophagy signaling. In the central nervous system, mTOR activity is regulated by nutrients, neurotrophic factors, and neurotransmitters that enhance protein synthesis and that activity of mTORC1 is imperative for neuronal growth, differentiation, and development^29^,^30^. Therefore, although autophagy can be achieved by downregulation of mTORC1 activity, disruption of mTORC1 signaling may also cause neurodegeneration and promote disease pathogenesis. In this study, we characterized two novel mTORC1-independent pathways, which are altered by Nox2-induced oxidative stress in PD pathogenesis. We demonstrated that oxidative stress from mild activation of Nox2 impairs autophagic flux by disrupting Beclin1-VPS34 interaction, a complex essential for autophagosome maturation. We have also characterized that oxidative stress from excessive activation of Nox2 deregulated autophagic flux by decreasing lysosomal activity.

Effects of neurotoxins such as rotenone, MPP^+ ^, and 6-hydroxydopamine (6-OHDA), have been shown to induce dopaminergic cell death *in vivo* and *in vitro*. Previous studies on the effect of these neurotoxins in PD models have suggested that all the neurotoxins do not render PD pathology through alterations of a single common pathway, rather the neurotoxins exhibit PD phenotypes through separate distinct mechanisms^31^,^32^. Although these neurotoxins induce Parkinsonian syndromes to a similar extent, vastly different effects of these neurotoxins on bioenergetics have been reported. Previous study has also shown that rotenone at different times of exposure differentially regulate autophagy in SHSY-5Y cells^33^. It has been shown that cells upregulate autophagy signaling in response to a very low dose of rotenone for 24 h of exposure, possibly as a bioenergetic adaptation to the metabolic stressor^33^. Similarly, we have also found that exposure to 0.5 μM rotenone for 24 h triggers autophagy signaling pathways. Moreover, we have shown that these effects are significantly regulated by Nox2 activity.

While DCF-DA is widely used as a probe for detecting intracellular ROS^34^,^35^, previous studies have established limitations of DCF-DA as there were several artifacts associated with the DCF-mediated ROS generation^36^,^37^,^38^. Therefore, to measure ROS generated from Nox2, we have used Nox2-specific redox sensor (p47-roGFP)^4^.

Previous studies have established Nox2 as a major source of ROS production, at least in early stages, in Huntington’s disease^39^,^40^. Valencia *et al.* have recently demonstrated a novel association between Nox2 and mitochondria, where Nox2-dependent ROS triggers mitochondrial-ROS generation and that inhibition of Nox2-mediated ROS abolished ROS generation from mitochondria^40^. These studies strongly suggest a possible involvement of Nox2 in several other neurodegenerative diseases. Future studies to characterize the cross-talk between Nox2 and mitochondria would greatly advance our understanding of the precise role of subcellular ROS in the pathophysiology of neurodegenerative disorders. In the present study, using Nox2-specific inhibitor, gp91 ds, we are able to quantify Nox2-dependent ROS generation in response to rotenone treatment and characterize the novel mechanisms by which Nox2-dependent oxidative stress leads to the pathogenesis of sporadic forms of PD. Taken together, our study highlights that proper modulation of NADPH oxidase and its downstream pathways might hold a potential therapeutic aspect for the treatment of Parkinson’s disease as well as other neurodegenerative diseases associated with increased oxidative stress and accumulation of toxic cellular constituents.

## Materials and Methods

### Reagents and plasmids

Rotenone (RT) was purchased from Sigma-Aldrich. DCFH-DA (6-carboxy-2′,7′-dichlorodihydrofluorescein diacetate). LysoTracKer red DND-99 was from Invitrogen. Phosphate buffered saline (PBS) was from GIBCO. The Nox-specific peptide inhibitor gp91 ds was from Biosynthesis, Lewisville, TX. LY294002 was purchased from cell signaling (9901 S). Torin1 was from Tocris Bioscience (CAS No: 1222998-36-8). Bafilomycin A was purchased from Sigma (B1793). Agarose beads were purchased from Roche. Mounting medium with DAPI was purchased from VECTASHIELD (Vector Laboratories, Inc. Burlingame, CA). WT-Src kinase and CA-Src kinase constructs were purchased from Addgene (Plasmid # 17672 and 13660 respectively). All antibodies used in this study are listed in Table S1 (Complete information about the concentration of antibodies used and companies from where we purchased).

### Cell culture and treatments

We have used the SHSY-5Y cell line, a very well-established and widely-used *in vitro* model of PD^1^,^41^,^42^, in this study. Previous studies have established that rotenone exposure to SHSY-5Y cells generates Parkinsonian phenotypes^1^,^41^,^42^. SHSY-5Y cells (ATCC® CRL2266™) were grown in DMEM-F12 (1:1, Gibco) supplemented with 10% heat inactivated fetal bovine serum (FBS, Atlanta Biologicals), 2 mM L-glutamine, 100 U/ml penicillin and 100 mg/ml streptomycin (Gibco). Treatment conditions are mentioned in results sections and figure legends as well.

### Live cell imaging by p47-roGFP

SHSY-5Y cells were seeded (2000/well) on 96-well plates for assessment of Nox2-specific ROS. Cells were transfected with our Nox specific redox biosensor p47-roGFP^4^. Transfection was done using X-tremeGENE HP DNA Transfection Reagent (Roche) at a ratio of 3:1 (Reagent to DNA). Changes in the redox sensor were assessed as we have previously described^4^.

### Autophagolysosome measurement by GFP-RFP-LC3 construct

The tandem fluorescent protein probe RFP-GFP-LC3^43^,^44^ was used to monitor autophagolysosome formation. In this probe, GFP signal is quenched in the acidic environment of the lysosome while RFP fluorescence remains unaffected during autophagolysosome formation. Impairment in delivery or fusion of the autophagosome to the lysosome leads to the accumulation of autophagosomes where GFP and RFP combined together to show yellow puncta of autophagosome. Confocal images were taken on a Zeiss LSM780 (Carl Zeiss) microscope with a 63x oil-immersion lens. GFP and RFP were excited at 488 nm and 568 nm, respectively. Fluorescence signals were monitored at 510 nm and 588 nm.

### MTT assay for cell survival

SHSY-5Y cells were grown and treated as indicated in figure legends. After treatment, cell proliferation was quantitated using a mitochondrial colorimetric assay (MTT assay, Sigma-Aldrich, St. Louis, MO) as per the manufacturer’s recommendations. The absorbance was measured at 570 nm and post-measurement corrected by subtracting absorbance at the reference wavelength of 690 nm. The results, expressed as relative optical density (OD).

### qRT-PCR for measurement of RNA level

SHSY-5Y cells were grown and treated as indicated in figure legends. After treatment , total RNA was extracted from cells using the RNEasy kit (Qiagen) according to the manufacturer’s instructions. One microgram was used for cDNA synthesis by QuantiTect Reverse Transcription kit (Qiagen). The primers for RT-PCR reactions are listed in Supplementary Table ST1. Quantitative real-time PCR was performed by using iQ SYBR Green Supermix on the CFX96 Touch Real-Time Detection System (Bio-Rad Laboratories). Samples were heated for 3 min at 95 °C and amplified in 39 cycles for 11 s at 95 °C, 45 s at 60 °C with last cycle of 10 sec at 95 °C, 5 s at 65 °C and 5 sec at 95 °C. Analyses were conducted using CFX manager software (Bio-Rad) and the threshold cycle (CT) was extracted from the PCR amplification plot. Relative gene expression was determined using the ΔΔCT method, normalizing to vehicle (DMSO). The change in mRNA levels of the genes was expressed in fold change as previously described^45^.

### DCFH assay for ROS measurement

Cells were seeded (2000/well) on 96-well plates for intracellular ROS measurements by DCFH-DA as suggested by the manufacturer’s protocol. 5 μm DCFH-DA dye was used as final concentration. DCF fluorescence was excited at 480 nm via a Sutter Lamda DG-5 Ultra high speed wavelength switcher, and emission intensity was collected at 510 nm.

### Immunobloting assay

After the end of treatment, cells were harvested and lysed in RIPA buffer (50 mM Tris-HCl, ph 7.4, 1% NP40, 0.5% Na-deoxycholate, 0.1% SDS, 150 mM NaCl, 2 mM EDTA, and 50 mM NaF) including a cocktail of protease (Roche) and phosphatase (SIGMA) inhibitors. Protein concentration was measured with the bicinchoninic acid (BCA) protein assay kit (Pierce, Rockford, IL), using BSA as standard. Lysates were separated via SDS-PAGE and then transferred to polyvinyldifluoride (PVDF) membranes. Blots were incubated in blocking buffer (5%, w/v, dried skimmed milk in Tris-buffered saline, pH 7.4, and 0.2% Tween 20, TBST) followed by overnight incubation with appropriate antibodies diluted in blocking buffer. Blots were then exposed to the IRDye® Secondary Antibodies (LI-COR) diluted in TBST for 90 min at room temperature and washed again. Blots were detected using LI-COR® Odyssey Infrared Imaging System and analyzed using ImageJ software. Complete detail of antibodies can be found in Supplemental Experimental Procedures.

### Immunoprecipitation assay

For co-immunoprecipitation, cells were lysed using NP-40 buffer (50 mM Tris-HCL (pH-7.5), 150 mM NaCl, 1% Np-40 (v/v) and 10% glycerol) for 30 mins at 4 °C. Equal amounts of protein lysates (200 μg) were pre-cleared for 30 mins at 4 °C on a nutator using 10ul protein-G agarose beads (Roche). Samples were centrifuged at 10,000 rpm for 1 min and equal amounts of antibodies (4ug/ul) were added to the pre-cleared supernatant together with 20ul of protein-G agarose beads. The samples were then incubated at 4 °C overnight on the nutator. Beads with immunocomplexes were centrifuged briefly at 3,000 g, washed three times in lysis buffer, and heated in laemmli SDS-sample buffer at 95 °C for 10 min. Samples were analyzed using immunoblot assay.

### Immunofluorescence assay

For immunofluorescence assay, 10,000 cells were grown on coverslips in 24-well plates. After the treatment, cells were rinsed with PBS once and fixed for 15 min with 4% paraformaldehyde in 1X PBS at room temperature (RT). The cells were then rinsed three times with PBS, and cells were permeabilized with 0.05% Triton X-100 in 1X PBS for 5 min. After rinsing twice with PBS, the cells were blocked with blocking reagent (0.1% saponin, 8% goat serum in PBS) for 1 h at RT. After washing once with PBS cells were incubated with primary antibody in 5% normal donkey serum for overnight at 4 °C. Cells were then rinsed four times with PBS, and incubated with secondary antibodies produced in donkey (diluted 1:600 in 5% normal donkey serum) for 1 h at room temperature in the dark. After that cells were washed four times with PBS and coverslips were mounted with vectashield containing DAPI (H-1200) prior to microscopy.

### Flow cytometry analysis

Cells were treated as described in figure legends and then kept in 200 nM acidotropic dye LysoTracker Red DND-99 (Molecular Probes) for 30 min. Lysosomal fluorescence of 10,000 cells per sample was determined by flow cytometry using the BD LSRFortessa™ Cell Analyzer (BD Biosciences) with the HTS auto-sampler device. See^45^ for detail information.

### Lysosomal hydrolases activity assay

The enzyme activity of lysosomal hydrolase TPP1 was measured by adapting a single-step intact cell assay protocol as described previously^46^. Briefly, SHSY-5Y cells were plated at a density of 20,000 cells per well in a 96-well plate (Corning, Inc.) and incubated at 37 °C and 5% CO_2_ overnight to achieve cell attachment. We plated four wells (three test wells and one background well) for each condition to be tested. The conditions for drug treatment were as follows: gp91 ds (5 μM) for 25 h, Rotenone (10 μM) for 24 h and a combined drug treatment in cells preincubated with gp91 ds (5 μM) for 1 h followed by 24 h of Rotenone (10 μM) treatment. The relative change in enzyme activity was measured by comparing each of the drugs to vehicle (Di-methyl sulfoxide-DMSO) treated wells at a concentration of 0.01%. After the treatment medium was removed and cells washed three times with PBS, the assay reaction was started by adding 125 μl of substrate solution to each well (0.25 mM Ala-Ala-Phe-7-amido-4-methylcoumarin-catalog no. A3401; Sigma in 0.1 M Na-acetate buffer pH 4.3, 0.5% Triton X-100 and 1 × protease inhibitor). Next, the released fluorescence was read from the bottom using a Synergy 2 plate reader (BioTek) at excitation 360/40 nm and emission 460/40 nm at time points 0, 4, 8 and 24 h. At any given time point, TPP1 enzyme activity was expressed as a fluorescence ratio between drug treated cells and vehicle (DMSO) treated cells after normalizing each well for its florescence reading at time (t-0).

### Data Analysis

Data are reported as mean ± SEM, unless otherwise specified. Statistical differences between groups were determined using ANOVA with Tukey’s post-hoc test. Statistical analysis was performed in Origin Pro (OriginLab Corporation, Northhampton, MA) with a significance level of *p < 0.05.

## Additional Information

**How to cite this article**: Pal, R. *et al.* NADPH oxidase promotes Parkinsonian phenotypes by impairing autophagic flux in an mTORC1-independent fashion in a cellular model of Parkinson’s disease. *Sci. Rep.* **6**, 22866; doi: 10.1038/srep22866 (2016).

## Supplementary Material

Supplementary Information
