# Supplementary material for: NADPH oxidase promotes Parkinsonian phenotypes by impairing autophagic flux in an mTORC1-independent fashion in a cellular model of Parkinson’s disease
Source: Sci Rep. 2016 Mar 10;6:22866. doi: 10.1038/srep22866 (PMC4785399; doi:10.1038/srep22866)
Supplement: Supplementary Information [file srep22866-s1.pdf]

**Supplementary materials**

NADPH oxidase promotes Parkinsonian phenotypes by impairing autophagic flux in an mTORC1-independent fashion in a cellular model of Parkinson's disease

Rituraj Pal[1], Lakshya Bajaj[2], Jaiprakash Sharma[2], Michela Palmieri[2], Alberto Di Ronza[2], Parisa Lotfi[2], Arindam Chaudhury[1], Joel Neilson[1,] Marco Sardiello[2] and George G. Rodney[1*]

[1]Department of Molecular Physiology and Biophysics, Baylor College of Medicine, Houston, United States.

[2]Department of Molecular and Human Genetics, Baylor College of Medicine, Houston, United States.

*To whom correspondence should be addressed.
Dr. George G. Rodney. Email: rodney@bcm.edu

**Supplementary figure legends**

**Supplementary Figure S1.** (**a**) SHSY-5Y cells were treated or untreated with 0.5 µM rotenone for 6 h. Some cells were preincubated with 5 µM gp91 ds for 1 h followed by 6 hour of rotenone treatment. As control, some cells were treated with 5 µM gp91 ds for 7 h or hydrogen peroxide ($H_2O_2$, 200 µM) for 15 min. ROS generation was measured by monitoring DCF-DA fluorescence intensity. DCF fluorescence is represented in RGB color scale using imageJ. (**b**) MTT assay was performed to measure cell-survivability in cells treated as (**a**). Bar diagram indicates quantitative analysis of at least 3 biological replicates. Results are represented as means of SE (SEM). $^{*\#,}p < 0.05$ versus all groups, unless otherwise indicated. N.S. indicates non-significant. (**c**) SHSY-5Y cells were treated or untreated with 0.5 µM rotenone for 6 h. Some cells were preincubated with 5 µM gp91 ds for 1 h or 5 mM NAC for 2 h followed by 6 hour of rotenone treatment. DMSO was used as control (vehicle). Cell lysates were analyzed by immunoblotting with antibodies as indicated. GAPDH was used as a loading control.

**Supplementary Figure S2.** (**a**) SHSY-5Y cells were treated or untreated with 0.5 µM rotenone for 24 h. Some cells were preincubated with 5 µM gp91 ds for 1 h followed by 6 hour of rotenone treatment. As control, some cells were treated with 5 µM gp91 ds for 25 h. Cells were labelled with endogenous beta-actin (green) and cathepsin D (red) for immunofluorescence. Representative cells are shown. The punctate structures indicate lysosomal localization of cathepsin D. Nucleus was labeled with DAPI (blue). (**b**) Cells were transfected with GFP-RFP-LC3 construct and incubated for 24 h, followed by treatment as indicated in (**a**). Live cell imaging was performed using confocal microscope. Bafilomycin was used as a positive control of autophagic flux blocker. Representative cells are shown where yellow pixels indicate colocalization of GFP and RFP in the merged images. Nucleus is indicated with DAPI (blue) staining. (**c**) MTT assay was performed to measure cell-survivability in cells treated as (**a**). DMSO was used as a control (vehicle).

Bar diagrams indicate quantitative analysis of at least 3 biological replicates. Results are represented as means of SE (SEM). N.S. indicates non-significant.

**Supplementary Figure S3.** (**a**) SHSY-5Y cells were treated or untreated with 10 µM rotenone for 24 h. Some cells were preincubated with 5 µM gp91 ds for 1 h followed by 24 hour of rotenone treatment. As control, some cells were treated with 5 µM gp91 ds for 25 h or hydrogen peroxide ($H_2O_2$, 200 µM) for 15 min. ROS generation was measured by monitoring DCF fluorescence intensity. DCF fluorescence is represented in RGB color scale using imageJ. (**b**) Lysates from cells treated as in (**a**) were analyzed by using immunoblot assay with the antibodies as indicated. (**c**) MTT assay was performed to measure cell-survivability in cells treated as (**a**). DMSO was used as a control (vehicle).

GAPDH was detected as a loading control for all immunoblots. Bar diagrams indicate quantitative analysis of at least 3 biological replicates. Results are represented as means of SE (SEM). $^{*}$p <0.05 versus all groups, unless otherwise indicated. N.S. indicates non-significant.

**Supplementary tables**

**Table ST1.**

**Sequences of oligos used in real-time qPCR analysis.**

| Gene name | Forward oligos | Reverse oligos |
|---|---|---|
| GAPDH | TGCACCACCAACTGCTTAGC | GGCATGGACTGTGGTCATGAG |
| SQSTM1 | AAGCTGCCTTGTACCCAC | CGCTCCGATGTCATAGTTCTTG |
| MAP1LC3B | AGCAGCATCCAACCAAAATC | CTGTGTCCGTTCACCAACAG |

**Table ST2.**

**Antibodies**

| NAME | COMPANY | DILUTION |
|---|---|---|
| LAMP1 | Cell signaling | 1:1000 |
| LAMP2 | Santa Cruz Biotechnology | 1:1000 |
| P62 | BD Bioscience | 1:1000 |
| LC3 (Immunofluorescence) | Novus Biologicals | 1:100 |
| LC3 (Immunoblot) | Cell signaling | 1:1000 |
| GAPDH | Millipore | 1:2000 |
| P-Src | Cell signaling | 1:500 |
| T-Src | Cell signaling | 1:1000 |
| P-PI3K | Cell signaling | 1:300 |
| T-PI3K | Cell signaling | 1:500 |
| P-Akt | Cell signaling | 1:500 |
| T-Akt | Cell signaling | 1:1000 |
| P-mTOR | Cell signaling | 1:500 |
| T-mTOR (Immunoblot) | Cell signaling | 1:1000 |

| | | |
|---|---|---|
| T-mTOR (Immunofluorescence) | Cell signaling | 1:50 |
| P-ULK1 | Cell signaling | 1:1000 |
| T-ULK1 | Cell signaling | 1:1000 |
| P-S6K | Cell signaling | 1:300 |
| T-S6K | Cell signaling | 1:1000 |
| P-4E-BP1 | Cell signaling | 1:1000 |
| T-4E-BP1 | Cell signaling | 1:1000 |
| P-S6 (S235/236) | Cell signaling | 1:1000 |
| P-S6 (S240/244) | Cell signaling | 1:1000 |
| T-S6 | Cell signaling | 1:2000 |
| P-Beclin1 | PhosphoSolutions | 1:50 |
| T-Beclin1 | Cell signaling | 1:1000 |
| P-AMPK | Cell signaling | 1:500 |
| T-AMPK | Cell signaling | 1:1000 |
| VPS34 | Invitrogen | 1:300 |
| Beta-actin (Immunofluorescence) | Cell signaling | 1: 200 |

| | | |
|---|---|---|
| Cathepsin D | Santa Cruz Biotechnology | 1:100 |
| PARP-1 | Cell signaling | 1:500 |
| Total caspase-3 | Cell signaling | 1:1000 |
| Cleaved caspase-3 | Cell signaling | 1:100 |

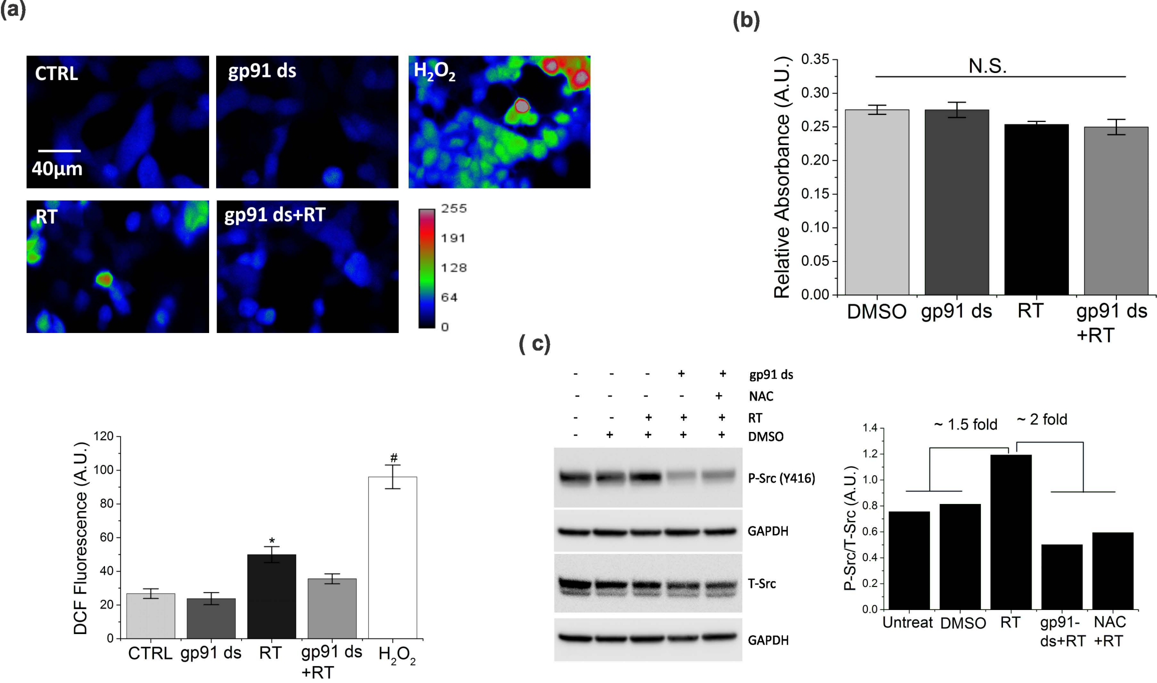
(a)
CTRL
gp91 ds
H2O2
40μm
RT
gp91 ds+RT
255
191
128
64
0
DCF Fluorescence (A.U.)
120
100
80
60
40
20
0
*
#
CTRL
gp91 ds
RT
gp91 ds +RT
H2O2
(b)
Relative Absorbance (A.U.)
0.35
0.30
0.25
0.20
0.15
0.10
0.05
0.00
N.S.
DMSO
gp91 ds
RT
gp91 ds +RT
( c)
gp91 ds
NAC
RT
DMSO
P-Src (Y416)
GAPDH
T-Src
GAPDH
P-Src/T-Src (A.U.)
1.4
1.2
1.0
0.8
0.6
0.4
0.2
0.0
~ 1.5 fold
~ 2 fold
Untreat
DMSO
RT
gp91-ds+RT
NAC +RT

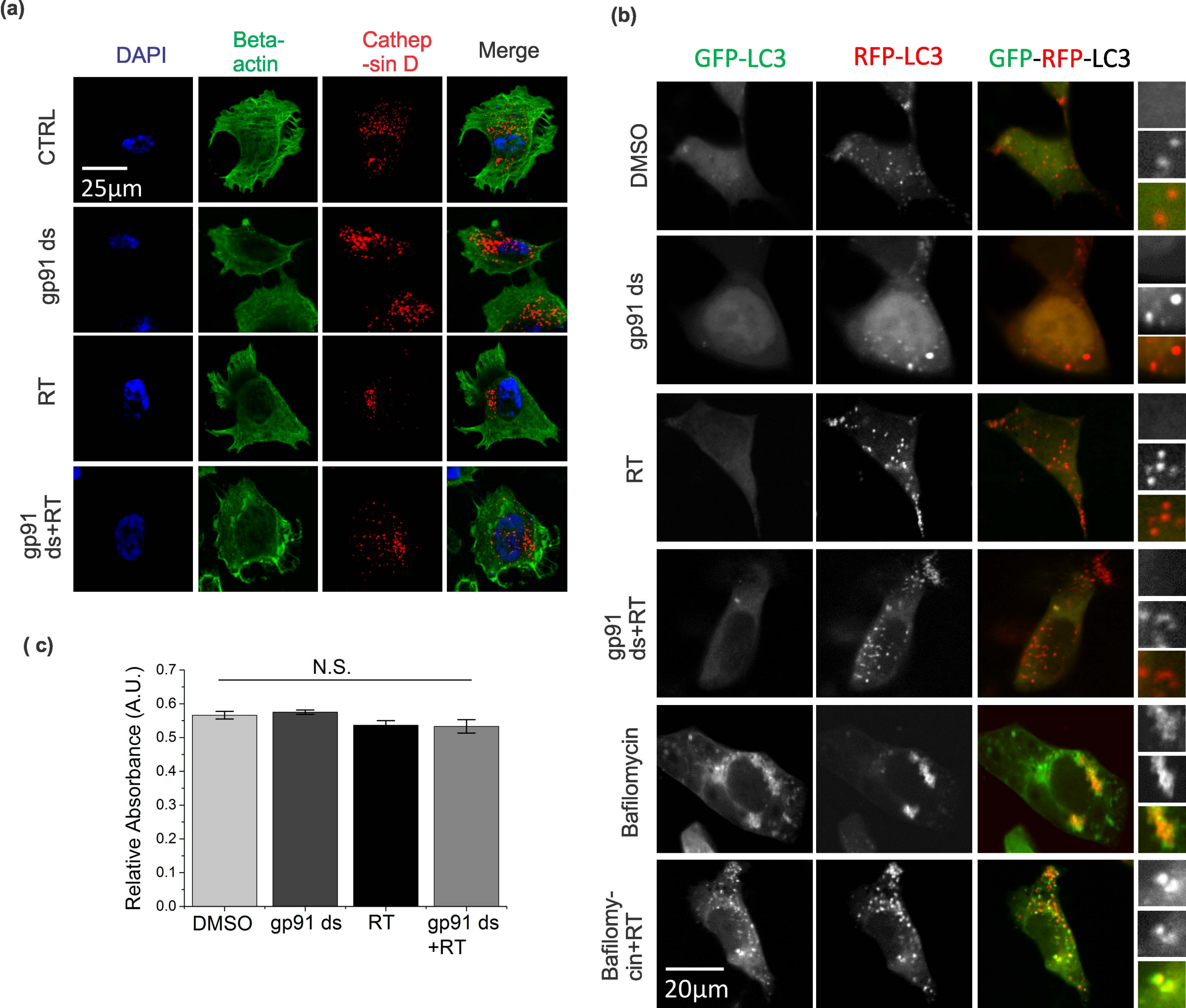

(a)
DAPI
Beta-actin
Cathep-sin D
Merge
CTRL
25μm
gp91 ds
RT
gp91 ds+RT
(b)
GFP-LC3
RFP-LC3
GFP-RFP-LC3
DMSO
gp91 ds
RT
gp91 ds+RT
Bafilomycin
Bafilomy-cin+RT
20μm
( c)
N.S.
Relative Absorbance (A.U.)
0.0
0.1
0.2
0.3
0.4
0.5
0.6
0.7
DMSO
gp91 ds
RT
gp91 ds +RT

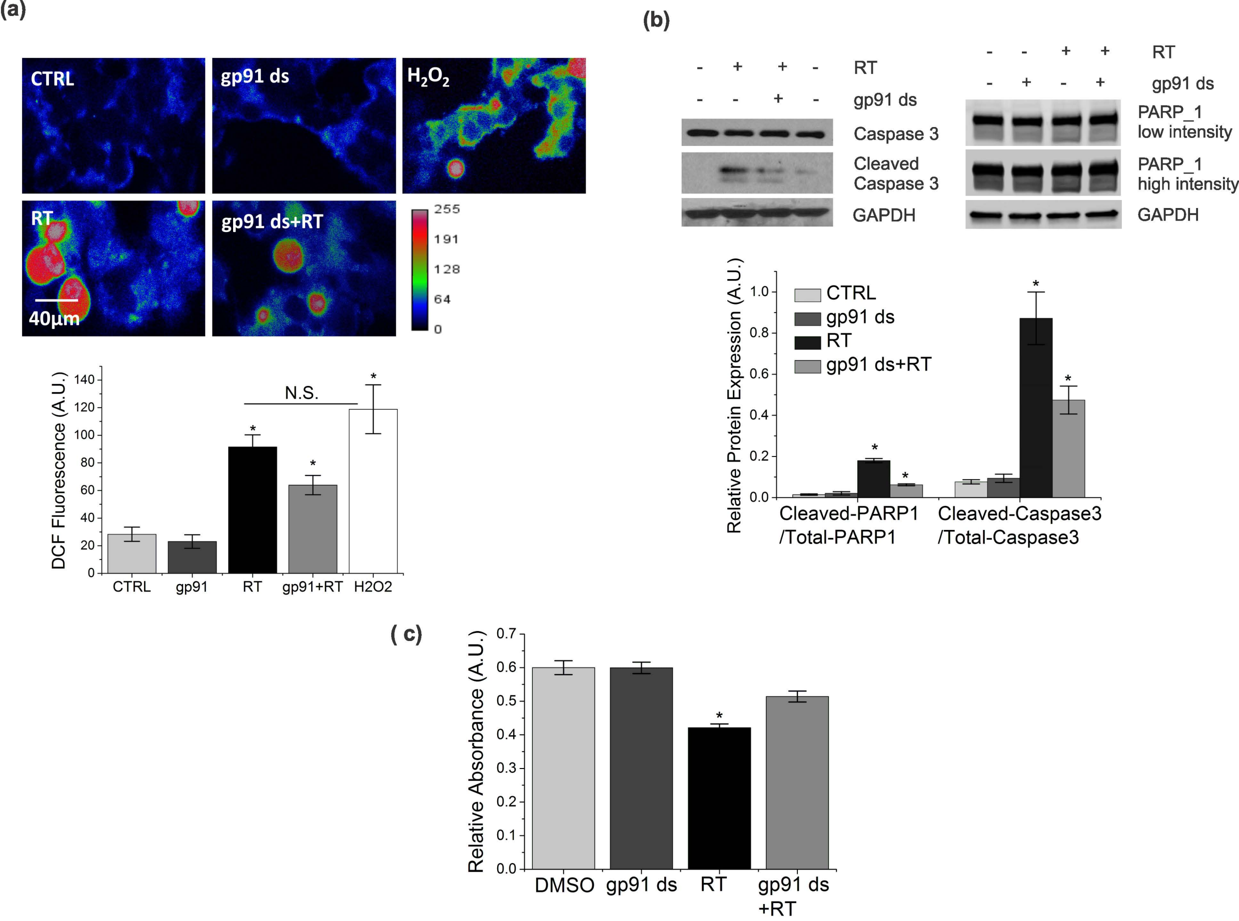

(a)
CTRL
gp91 ds
$H_2O_2$
RT
gp91 ds+RT
40μm
255
191
128
64
0
DCF Fluorescence (A.U.)
N.S.
CTRL
gp91
RT
gp91+RT
H2O2
(b)
RT
gp91 ds
Caspase 3
Cleaved Caspase 3
GAPDH
PARP_1 low intensity
PARP_1 high intensity
GAPDH
Relative Protein Expression (A.U.)
CTRL
gp91 ds
RT
gp91 ds+RT
Cleaved-PARP1 /Total-PARP1
Cleaved-Caspase3 /Total-Caspase3
( c)
Relative Absorbance (A.U.)
DMSO
gp91 ds
RT
gp91 ds +RT
